# Bioética y el derecho de acceso a los cuidados de salud

**DOI:** 10.18294/sc.2023.4491

**Published:** 2023-09-07

**Authors:** Volnei Garrafa

**Affiliations:** 1 Doctor en Ciencias, Posdoctorado en Bioética. Profesor Emérito, Centro Internacional de Bioética e Humanidades, Faculdade de Ciências da Saúde, Universidade de Brasília, Brasilia, Brasil. garrafavolnei@gmail.com Universidade de Brasília Centro Internacional de Bioética e Humanidades Faculdade de Ciências da Saúde Universidade de Brasília Brasilia Brazil garrafavolnei@gmail.com

**Keywords:** Derechos Humanos, Acceso a la Atención Médica, Bioética, Justicia, Equidad, Human Rights, Access to Health Care, Bioethics, Justice, Equity

## Abstract

El acceso a los cuidados de salud es un derecho universal de todo ser humano y no debe ser reducido a un objeto de consumo disponible solo para las personas con capacidad económica de adquirirlo. Desde el referencial teórico de la Declaración Universal sobre Bioética y Derechos Humanos de la Unesco, en primer lugar, se discute la razón de elegir los derechos humanos como base para el trabajo; en segundo lugar, se presenta la equidad como un principio indispensable en el debate para reforzar la comprensión de que las personas y las poblaciones desiguales deben ser tratadas de manera desigual y compensatoria, con el objetivo de buscar la igualdad basada en el reconocimiento humanitario del derecho de cada individuo en función de sus necesidades y diferencias. En tercer lugar, el texto aborda el complejo problema de priorizar la asignación de recursos escasos para garantizar el acceso a la atención médica para la mayor cantidad posible de personas. En resumen, el artículo pretende mostrar que el acceso a la atención médica para todas las personas, independientemente de su nivel de ingresos, debe ser considerado como un derecho humano universal que, además de las responsabilidades gubernamentales y del sector privado en apoyar programas inclusivos, debe reconocer también la legitimidad de la lucha de los movimientos sociales en defensa de mejores condiciones de vida y salud para todas las personas sin distinción.

## Abstract (en)

Access to health care is a universal human right and therefore should not be treated as a commodity only accessible to people with the economic means to acquire it. This study adopts a theoretical framework based on UNESCO’s Universal Declaration on Bioethics and Human Rights. The discussion first explores the rationale for choosing human rights as a foundation for such an endeavor. Secondly, the notion of equity is presented as an indispensable principle that should be incorporated into such discussions, reinforcing the understanding that unequal people and populations must be treated in a differentiated and compensatory manner, with the aim of seeking true equality based on the humanitarian recognition of every individual’s rights, accounting for their needs and differences. Thirdly, the text seeks to address the complex problem of prioritizing the allocation of scarce resources in order to ensure access to health care for as many people as possible. In summary, this article intends to demonstrate that access to health care for all people, regardless of their income level, should be considered to be a universal human right. Beyond the obligations of governments and the private sector to support inclusive programs, there is also a need to acknowledge social movements’ legitimate struggles for achieving better living conditions and health outcomes for all people, without exception.

## INTRODUCCIÓN

El estudio del acceso a los cuidados de salud puede ser abordado desde diversos ángulos y perspectivas. Para analizar este tema, es necesario establecer inicialmente un enfoque teórico y aplicado para su abordaje. Desde una visión macroscópica del asunto, surgen dos cuestiones principales que deben enfrentarse. Una de ellas se refiere al tipo de atención médica al que se desea hacer referencia: ya sea la atención primaria (básica o de promoción de la salud), la secundaria (específica, especializada o terapéutica propiamente dicha) o la atención integral, que engloba ambas formas de atención. La otra cuestión, más compleja, se relaciona con el concepto polémico presente en el contexto contemporáneo de la mayoría de los países, acerca de lo que se entiende en la práctica concreta por “acceso a los cuidados de salud”. Si este acceso se considera como un bien de consumo, es decir, como una mercancía similar a otros objetos que se ofrecen en el libre mercado a las personas usuarias para ser comercializado según las reglas de oferta y demanda. O si, por el contrario, se interpreta como un derecho humano universal que surge desde el nacimiento de la persona y es responsabilidad pública de los Estados y/o de la solidaridad de individuos o colectivos hacia sus semejantes de la especie Homo sapiens, y al cual todas las personas, sin distinción, deberían tener acceso.

Ante la situación presentada, el objetivo es enfocar el acceso a los cuidados de salud en su integralidad, es decir, desde las acciones más básicas hasta las más complejas, incluyendo no solo lo que se entiende por “acceso a los cuidados de salud” o “acceso a la asistencia en salud”, sino también el “acceso a la salud” en sí mismo, en su amplio espectro, no solo biológico, sino también social (en el sentido de la universalidad de acceso para la inclusión de las personas en el sistema de atención), económico (en términos de costos) y político (en términos de definición de prioridades gubernamentales en sus políticas públicas).

En cuanto a la fundamentación teórica que se utiliza en el desarrollo posterior del trabajo, se basa en la Declaración Universal sobre Bioética y Derechos Humanos de la UNESCO^1^, que en su Artículo 14 sobre la “Responsabilidad Social y Salud”, establece que:

“1. La promoción de la salud y el desarrollo social para sus pueblos es un cometido esencial de los gobiernos, que comparten todos los sectores de la sociedad. 2. Teniendo en cuenta que el goce del grado máximo de salud que se pueda lograr es uno de los derechos fundamentales de todo ser humano sin distinción de raza, religión, ideología política o condición económica o social, los progresos de la ciencia y la tecnología deberían fomentar: a) el acceso a una atención médica de calidad y a los medicamentos esenciales, especialmente para la salud de las mujeres y los niños, ya que la salud es esencial para la vida misma y debe considerarse un bien social y humano”.

Además, también se utilizará el Artículo 15 de la misma Declaración, que trata del “Compartir beneficios”, el cual establece que los beneficios derivados de la investigación científica deben contemplar también el “b) acceso a la atención médica de calidad” y el “d) apoyo a los servicios de salud”.^1^

Basándonos en estos dos artículos de la Declaración, además del Artículo 10 que trata de “Igualdad, justicia y equidad”, se partirá de la comprensión de que el acceso a los cuidados de salud es un derecho universal de todo ser humano, de manera que las acciones necesarias para su logro puedan ser disfrutadas plenamente por las personas, sin reducirse a simples objetos de consumo adquiridos en el mercado de la vida únicamente por individuos o grupos con recursos financieros para ello.

Siguiendo esta línea de ideas, la presentación del tema se divide en tres grandes subtemas. En primer lugar, se analiza la razón detrás de la elección del enfoque de los Derechos Humanos como hilo conductor del estudio sobre la universalización del acceso a la atención médica, desarrollando algunos conceptos que permitan una mejor comprensión de las bases que sustentan las ideas presentadas. En segundo lugar, se busca dejar claro que no tiene sentido hablar sobre bioética global en los libros académicos si no se tiene la valentía de abordar temas espinosos como los altos costos asociados con el acceso a la atención médica y la atención integral a la salud de las personas en este comienzo del siglo XXI. En tercer lugar, se incorpora al debate un principio indispensable: la equidad, entendida como la necesidad de tratar compensatoriamente de manera desigual a personas y poblaciones desiguales, con el objetivo (aún distante a nivel global, pero que debe perseguirse incansablemente) de buscar y alcanzar la verdadera igualdad, basada en la disposición humanitaria de reconocer igualmente el derecho de cada individuo, partiendo de sus necesidades y diferencias. Para concluir, el capítulo aborda la cuestión no menos compleja y espinosa relacionada con la priorización en la asignación de recursos, especialmente escasos en los países periféricos, para lograr un creciente acceso a la atención médica para la mayor cantidad posible de personas que lo necesitan.

## EL ACCESO A LOS CUIDADOS DE SALUD COMO UN DERECHO HUMANO

En el año 1978, en la ciudad de Alma Ata, Kazajistán, la Asamblea de la Organización Mundial de la Salud, reunida en esa ocasión, definió “Salud para todos en el año 2000” como una meta a alcanzar. Aunque a primera vista esta frase podría parecer una utopía inalcanzable, estimuló a naciones industrializadas y países empobrecidos por igual a reflexionar con mayor objetividad sobre la situación sanitaria mundial de ese tiempo y sobre el derecho de las personas y pueblos de los cinco continentes al acceso universal a la salud y a los cuidados básicos inherentes a ella.

El brasileño Josué de Castro, presidente del Consejo Ejecutivo de la Organización de las Naciones Unidas para la Alimentación y la Agricultura (FAO) entre 1952 y 1956, escribió en el ya lejano año de 1946 el inquietante libro *Geografía del hambre*^2^, complementado poco después por el no menos incómodo *Geopolítica del hambre*^3^, develando el mapa del flagelo que afectaba a una gran parte de la población en ese momento^4^. Pasados cerca de 70 años de esas publicaciones, a pesar de los enormes avances registrados en el campo científico y tecnológico, lamentablemente ese panorama indeseado ha cambiado muy poco.

En el campo específico de la salud, con temas aparentemente tan alejados como el hambre, mencionado anteriormente -y más recientemente la obesidad, para contrastar de manera dialéctica y cruda la situación-, las cosas tampoco han ocurrido de manera diferente. Por un lado, los países enriquecidos, junto con algunos sectores privilegiados de población en los países periféricos, han experimentado avances extraordinarios en el sector, proporcionando acceso a nuevas técnicas, insumos y medicamentos, entre otros aspectos y actividades, añadiendo varios años de vida de calidad a sus habitantes. Por otro lado, en una situación diametralmente opuesta, un número incontable de personas en los países periféricos, especialmente en el hemisferio sur del mundo, siguen sufriendo y muriendo de las mismas enfermedades que sus padres y abuelos sufrían y morían en generaciones anteriores, con las estadísticas internacionales mostrando solo ligeras variaciones en sus perfiles epidemiológicos.

Mientras que la expectativa de vida al nacer en Europa Occidental, América del Norte o Japón superó los 80 años a principios del siglo XXI, en muchos países africanos como Burkina Faso y Sierra Leona, entre otros, apenas llega a los 40 años. En otras palabras, individuos de la misma especie Homo sapiens, con la misma programación genómica, por haber tenido el privilegio de nacer en un lugar “mejor”, disfrutarán el doble de tiempo de vida que otros que no tuvieron la misma suerte. La afirmación que se deriva de esta reflexión, que es real, concreta y actual, es que: los países desarrollados exigen globalmente el cumplimiento de ideales democráticos y derechos humanos; la producción mundial de alimentos es suficiente para garantizar que los ocho mil millones de habitantes del planeta puedan comer tres veces al día; la economía acumulada supera ampliamente la capacidad de proporcionar trabajo, transporte y vivienda digna para permitir que las personas y los pueblos sobrevivan con un mínimo de bienestar y dignidad. El problema, pués, no es “lo que dicen defender”, sino el acceso y la distribución justa y equitativa a las personas, de los bienes de consumo producidos.

Las consecuencias de esta situación se manifiestan en las precarias condiciones de vida de estas personas, haciéndolas vulnerables y enfermas de forma prematura, e incluso sin acceso a los cuidados mínimos necesarios para atender su salud. La concentración de recursos y suministros en algunos lugares, en contraste con la desigualdad e inaccesibilidad en otros, sigue siendo abrumadora. La inversión de unas pocas decenas de dólares por persona al año en muchos países africanos demuestra que la escasez de recursos para la atención de la salud es una realidad que hace que la inversión esté muy por debajo de lo estipulado como mínimo necesario por la Organización Mundial de la Salud (OMS).

Además de la escasez de recursos públicos para invertir en salud en los países periféricos, la extrema pobreza sigue siendo uno de los aspectos que más vidas cobran en el mundo globalizado del siglo XXI, por más paradójico que esto pueda parecer. Ha pasado más de medio siglo desde que el hombre llegó a la luna y hemos entrado en la era de los trasplantes múltiples de órganos, la nanotecnología, la terapia celular y la inteligencia artificial, pero este aspecto crucial persiste. Esto no puede ser pasado por alto en cualquier discusión responsable que relacione los derechos humanos universales y el acceso a la atención médica.

Sin embargo, si el derecho a la salud aborda cuestiones fundamentales de la justicia social, el incumplimiento del derecho a la atención médica, es decir, a la asistencia sanitaria, también puede verse como consecuencia de la falta de priorización de medidas gubernamentales de atención pública y de ineficiencias administrativas. Es a través de la exigencia sociopolítica de cumplir el derecho a la salud que los países y los pueblos podrán abordar todas estas cuestiones repetidamente señaladas en diferentes informes mundiales sobre la salud en el mundo. Situaciones como la exclusión social y la extrema pobreza, por lo tanto, no solo impiden el cumplimiento del derecho social e histórico a la atención médica, sino que obstaculizan especialmente el cumplimiento del derecho humano más fundamental: el derecho a la vida misma, a través de uno de sus requisitos primarios, el derecho a la salud.

Aunque los problemas son muy diferentes, los países industrializados y desarrollados también tienen sus desafíos que enfrentar y resolver. Mientras que, para las naciones más empobrecidas, la cuestión central es decidir dónde recortar recursos ante situaciones diarias de escasez económica, en las naciones más enriquecidas, las discusiones generalmente giran en torno a prioridades y, en el mejor de los casos, en algunas medidas prácticas de racionalización de gastos. Sin embargo, un elemento nuevo que ha surgido en las últimas décadas y que ha sido objeto de críticas por parte de algunos estudiosos más agudos es el relacionado con los altos costos derivados del uso de nuevas tecnologías médicas, que están generando dificultades casi insuperables para el mantenimiento de los sistemas públicos y de seguridad social de atención médica, incluso en los países desarrollados.

Daniel Callahan, cofundador y durante muchos años presidente emérito del Hastings Center de Nueva York, una institución progresista de referencia en el campo de la bioética, fue un crítico constante de situaciones como las descritas anteriormente a lo largo de toda su larga trayectoria profesional y académica. El autor siempre consideró los límites impuestos en el ámbito de la salud como un “insulto” a la medicina y al inexorable proceso de la “finitud humana”, un proceso que, según él, ya contaba a finales del siglo pasado con poderes enormes para mitigarlo a través de nuevas posibilidades terapéuticas que brindan renovadas esperanzas^5^^,^^6^. Callahan, quien falleció en 2019, fue uno de los raros estudiosos estadounidenses que defendió de manera constante e insistente una salud pública y universal que recurriera a medidas de racionalización en caso de recursos insuficientes. Pero iba más allá: para él, tales medidas de racionalización deberían ser debatidas públicamente y definidas con claridad por los gobiernos, antes de aceptar simplemente la marginación de las personas empobrecidas y la pérdida de confianza en el personal de salud; quienes, por las propias circunstancias, se ven obligados a participar en actividades que, según muchos investigadores radicados en el sur geopolítico, son éticamente cuestionables^7^.

En una de sus últimas publicaciones, Callahan^8^, haciendo una especie de “balance” de su vida profesional, reafirmó sus convicciones, enfatizando que:

“Después de unos 30-40 años de esfuerzos e investigaciones gubernamentales y privadas a nivel internacional, y después de gastar miles de millones de dólares, la situación está empeorando cada vez más, con poco progreso realmente serio. Quizás haya cierto masoquismo (o incluso arrogancia) de mi parte al abordar un tema tan difícil y a menudo deprimente, pero también había otra razón para la elección: en todos los casos, hay un consenso en que abordar el problema requiere cambios profundos en la estructura ya arraigada en diferentes culturas, comportamientos y formas de vida”.^8^

## IGUALDAD, JUSTICIA, EQUIDAD Y ACCESO A CUIDADOS DE SALUD

La Declaración Universal sobre Bioética y Derechos Humanos de la UNESCO también incorporó el término “equidad” a su conjunto de principios, al incluirlo en su Artículo 10 ya mencionado, en conjunción con los temas de “igualdad” y “justicia”, con el siguiente contenido: “La igualdad fundamental entre todos los seres humanos en términos de dignidad y derechos debe ser respetada de manera que todos sean tratados de manera justa y equitativa”^1^. Hacemos referencia a este conjunto de principios, especialmente para enfatizar la “interrelación y complementariedad de los principios” propuesta en el Artículo 26 de la propia Declaración, lo que le brinda aún más consistencia y armonía: “La presente Declaración debe considerarse en su totalidad y sus principios deben entenderse como complementarios e interrelacionados. Cada principio debe interpretarse en el contexto de los demás, de manera pertinente y adecuada a cada circunstancia”^1^.

Nunca está de más recalcar que la interpretación de la justicia en la bioética no es la misma que se da al tema en el ámbito del derecho y la filosofía. Del mismo modo en que con el tiempo se ha construido una sólida fundamentación teórica y operacional actualmente existente en el campo de la promoción de la salud y/o la atención primaria, hoy en día es indispensable repensar el principio de justicia en bioética con otros referentes, que vayan más allá de las contradicciones simplistas y directas representadas por conflictos binarios como bien-mal, justo-injusto, correcto-incorrecto y, especialmente, inclusión-exclusión. En un estudio desarrollado en este sentido, se buscó diferenciar, sin separar, la visión de la justicia de los límites del legalismo jurídico exclusivo, con el objetivo de ampliar y profundizar la epistemología del tema en relación con el campo de la bioética y las cuestiones relacionadas con la justicia como un derecho humano^9^. En dicho trabajo, la justicia, como principio indispensable de una bioética pluralista, laica y orientada hacia los derechos humanos universales, requiere más fuertemente un carácter de legitimación moral que de legalismo jurídico. En este sentido, según sus autores:

No está de más enfatizar que el trabajo de la bioética no está dirigido principalmente hacia el tema de los límites, es decir, aquellas cosas que pueden o no pueden hacerse, aquellas cosas que son correctas o incorrectas desde el punto de vista del principio de justicia. Lo que importa para una bioética justa, democrática y ciudadana son las razones que justifican un determinado juicio de valores y la correcta aplicación de las decisiones.^9^

Considerando que el derecho de las personas a tener acceso a la atención médica constituye un valor afirmativo de referencia en el contexto de la bioética, la pregunta que surge entonces es: ¿cómo llevar este justo derecho a la práctica? Y es precisamente, en este sentido, que se busca incluir en esta discusión, además del tema de la justicia, reflexiones sobre equidad e igualdad.

Aunque con diferentes denominaciones, las referencias a la equidad se remontan a la antigua Grecia, pasando por el Derecho Romano y la Edad Media, hasta llegar finalmente a los tiempos actuales, a partir del período posterior a la Segunda Guerra Mundial. En este tiempo más reciente, comenzó a aplicarse sobre todo en el campo de la justicia, recibiendo diversas interpretaciones. Los movimientos sociales, en especial aquellos involucrados en la lucha contra las diversas formas de discriminación por género, raza y sexo, fueron sus principales precursores. Más recientemente, a partir de la década de 1970, el concepto empezó a adaptarse e incorporarse cada vez más al ámbito de la salud, especialmente en las discusiones relacionadas con la toma de decisiones sobre la distribución de recursos escasos en el campo de la salud pública.

En una reunión celebrada en Ginebra en 1996, cuyo objetivo fue revisar los objetivos de la OMS en relación con la propuesta de “Salud para Todos en el Año 2000”^10^, el concepto de equidad cobró fuerza, convirtiéndose definitivamente en una palabra clave en el campo de la salud pública y colectiva a finales del siglo pasado. Básicamente, la equidad significa estar dispuesto a reconocer el derecho de cada individuo en función de sus diferencias.

En esta línea de reflexión, es oportuno centrar la atención en la posibilidad de aplicar criterios justos para la distribución de la atención médica, desde los conceptos de justicia y equidad. A partir de la propuesta de un modelo de justicia basado en los fundamentos primarios de la sociedad, John Rawls^11^ desarrolló una teoría relacionada con el principio de justicia que, según él, debería estar presente en las instituciones públicas y privadas, ya que serían intermediarias entre las personas en la convivencia social. El autor parte de la idea de que la distribución de recursos debe ocurrir en dos etapas. En la primera, la preocupación sería la distribución igualitaria de derechos y deberes básicos. En la segunda, a partir del principio de la diferencia, se compensarían las desigualdades injustas, asegurando igualdad de oportunidades para todas las personas. Su enfoque fue la distribución de los bienes primarios sociales esenciales, sin incluir la salud, que él interpretó como un “bien primario natural”. Norman Daniels^12^ trabajó sobre la teoría de Rawls, extendiéndola a la salud en su amplio sentido, otorgándole un estatus moral especial, sustentado en los principios de libertad, diferencia e igualdad de oportunidades. Aunque ninguno de los dos autores logró una respuesta definitiva sobre la distribución justa de recursos, sus ideas estimularon reflexiones útiles sobre la necesidad de reducir las injustas desigualdades en el campo de la salud, así como fomentar discusiones sobre cooperación social, libertad, bases de igualdad, asignación de recursos escasos, distribución adecuada de ingresos, riqueza y oportunidades^13^.

Al igual que en la Declaración, al incorporar el tema de la igualdad en el debate sobre justicia y equidad, la igualdad se entiende como la consecuencia deseada de la equidad, siendo esta el punto de partida. Es decir, solo a través del reconocimiento de las diferencias y las diversas necesidades de los sujetos sociales se puede alcanzar la igualdad. La igualdad ya no sería un punto de partida ideológico abstracto que tiende a anular las diferencias, sino que se convierte en el punto de llegada de la justicia social, un referente de los derechos humanos donde la consecuencia natural lleva al reconocimiento de la ciudadanía^14^.

La equidad se convierte, entonces, en la base ética que guía el proceso de toma de decisiones en la asignación de recursos para optimizar el acceso a la atención médica. A través de la utilización de este principio, junto con los principios de responsabilidad (individual y pública) y justicia, es posible que el derecho a la salud se acerque a la realidad. La equidad, es decir, el reconocimiento de necesidades diferentes de sujetos también diferentes para lograr derechos iguales, es uno de los caminos de la ética práctica en relación con la realización de los derechos humanos universales, incluido el derecho a la vida, representado en este análisis por la posibilidad de que las personas reciban atención médica según sus necesidades. La equidad es, por lo tanto, un principio teórico-práctico que permite abordar al menos parte de las distorsiones en el acceso a la atención médica, al proporcionar condiciones para mejorar la calidad de vida de segmentos significativos de la población.

Establecer prioridades en el campo específico del acceso a la atención médica, una de las cuestiones centrales del debate sobre la ética de la asignación de recursos en la salud pública o incluso privada, implica jerarquizar las necesidades humanas. En este sentido, los administradores -especialmente los públicos, encargados de tomar las decisiones que se pondrán en práctica- deben evitar caer en extremos perniciosos que ya ocurren en algunos lugares en los que, por ejemplo, comités especiales establecen de manera lineal listas de prioridades en la atención médico-hospitalaria sin considerar cada caso o situación específica, basándose en criterios altamente discutibles relacionados con políticas clientelistas locales y completamente alejadas de la ética. No es aceptable, por ejemplo, colocar a un ciudadano alcohólico (y, por lo tanto, enfermo) en el último lugar de las listas de espera para un trasplante hepático de manera horizontal y acrítica.

Por otro lado, no se puede pasar por alto en el contexto de esta discusión los altos costos que han alcanzado los tratamientos médicos en las últimas décadas, que incluyen innumerables pruebas de laboratorio y otros exámenes no siempre indispensables, además de intervenciones delicadas y costosas, lo que resulta en la inaccesibilidad para un número creciente de personas. Situaciones como estas han llevado a algunos investigadores a plantear interrogantes inquietantes sobre el futuro en lo que respecta a los beneficios reales de los avances y descubrimientos, como ya se mencionó anteriormente: si serán un bien común o un privilegio para unos pocos. En este sentido, Montagnier^15^ ya señalaba hace más de 30 años una perspectiva de difícil solución:

“La investigación médica nos llevará a soluciones extraordinarias, pero que serán tan caras que ocasionarán tremendas deudas sociales. Las terapias preventivas que podrán aplicarse antes de la aparición de lesiones irreversibles quizás logren prolongar la vida promedio en veinte años más [...] Pero las consecuencias sociales de esta revolución biológica serán impredecibles. Es claro que no podrán beneficiar a diez mil millones de individuos y que los beneficios solo serán accesibles para aquellos que tengan los medios para pagarlos”.^15^

La preocupación por este problema crece a medida que las cosas se desarrollan a nivel internacional. La incapacidad para conciliar fines contradictorios entre sí, como la racionalización y contención de costos, la calidad de la atención, la igualdad en el acceso a los servicios de salud y la libre elección de quién brinda y recibe el tratamiento, exige una reflexión incluso por parte de aquellas personas que siempre han defendido el derecho universal a la salud y a la atención médica^16^. Tanto desde el punto de vista cultural y moral como en relación con sus efectos prácticos, todos los derechos mencionados anteriormente fueron tesis predominantes durante gran parte del siglo XX, cuando la teoría del Estado de bienestar fue un tema recurrente en varios países, especialmente en Europa Occidental^17^.

Según Sen y Klisberg^18^, la equidad incluye cuestiones directamente relacionadas con el logro de la salud y con la capacidad misma de generar una buena salud, no solo considerando la distribución propiamente dicha de la atención médica y las formas de acceso a ella, sino también incorporando la justicia procesal y asociando, de esta manera, la importancia de la no discriminación en la prestación de los servicios de salud. Además, según ellos, el compromiso con la equidad en salud debe incluir temas más amplios del ámbito de la justicia social y de la equidad en general, sin perder de vista la aplicación más flexible de recursos y los posibles logros e impactos que pueden alcanzarse a partir de diversos arreglos sociales inclusivos y políticamente factibles de construir.

## ACCESO A LA ATENCIÓN MÉDICA Y PRIORIZACIÓN EN LA ASIGNACIÓN DE RECURSOS

En la actualidad, ya no es posible seguir considerando los preceptos y valores como variables derivadas exclusivamente de lo emotivo o lo individual; o como solía decirse antiguamente, como aspectos “superestructurales”. Las cuestiones éticas, en prácticamente todos los campos de la actividad humana, en gran parte de los países del actual mundo globalizado, han adquirido en términos históricos connotaciones públicas, dejando de ser meros problemas de conciencia individual que se resuelven en la esfera privada y en el ámbito íntimo.

Este enfoque no pretende entrar en conflicto con el discurso de la autonomía. Por el contrario, busca evitar la connotación maximalista que este importante principio bioético ha adquirido, sobre todo a partir de algunas voces académicas de cultura anglosajona que, con cierta frecuencia, lo llevan deliberadamente hacia la individualidad, que a su vez puede deslizarse hacia el individualismo y caer en un inevitable egoísmo, en su mayoría incompatible con la implementación de políticas públicas (e incluso privadas) moralmente justas y políticamente equilibradas que busquen el bien común.

Hans Jonas^19^ fue uno de los autores que más se dedicó en el siglo XX a cuestiones relacionadas con la ética de la responsabilidad. Según su línea de pensamiento, en lo que respecta al campo de la ciencia y el derecho de acceso a ella, por ejemplo, la libertad de creación y el uso de nuevos conocimientos deben estar relacionados con la responsabilidad, tanto individual como pública, en la aplicación de los descubrimientos y sus consecuencias.

En lo que respecta a la “ética de la responsabilidad pública”, un aspecto que no debe pasarse por alto en la reflexión sobre la salud es la definición de las prioridades en las inversiones del Estado, incluido el estudio de la asignación, distribución y control de los recursos financieros destinados al sector. A diferencia de los países industrializados, las naciones periféricas enfrentan situaciones paradójicas que van desde la persistente presencia de enfermedades comunes de la pobreza (dengue, malaria, Chagas, esquistosomiasis, fiebre amarilla, etc.) hasta un registro significativo en las estadísticas de mortalidad de cuestiones que también ocurren con frecuencia en países más avanzados (cáncer, problemas cardiovasculares, accidentes cerebrovasculares, accidentes de tráfico, etc.). Con los altos costos de los medios de diagnóstico y la creciente sofisticación tecnológica, los recursos invertidos en salud comienzan a resultar insuficientes incluso en los países enriquecidos del hemisferio norte.

La discusión sobre “prioridades”, por lo tanto, está adquiriendo connotaciones éticas cada vez más dramáticas. Es responsabilidad del Estado y de las instituciones públicas y privadas encontrar soluciones morales para enfrentar la escasez, soluciones que no impliquen discriminación injusta ni la tiranía de minorías^20^. Sobre todo en el contexto de los países más empobrecidos, la expresión “encontrar soluciones morales individualizadas” o “priorizar recursos públicos” debe significar “brindar atención preferencial a la mayoría de la población necesitada”. Este es un aspecto que debe abordarse desde la comprensión y la aplicación del enfoque de la equidad.

La cuestión de la asignación y distribución de recursos en salud, por lo tanto, está adquiriendo cada vez más importancia política y social. Está directamente relacionada con la determinación de las prioridades de inversión del Estado y con la decisión ética de cuánto se destinará del presupuesto global al sector, una decisión que es inevitablemente política^21^. Además de esto, es importante analizar el cumplimiento de las disposiciones legislativas con respecto a los porcentajes destinados a la salud en cada país, así como la prioridad que deben recibir algunas iniciativas en relación con otras, como se discutió anteriormente.

Las decisiones éticas a menudo implican elecciones que conllevan la selección de grupos de la humanidad que serán beneficiados prioritariamente o no. Esto también se aplica a la definición de las prioridades en la asignación de recursos escasos para la salud. Aunque sea una tarea dramática, debe seguir realizándose. El objetivo de esta línea de reflexión es simplemente señalar que es necesario tener una base ética que oriente el ejercicio de las decisiones en casos de necesidad de priorización en la asignación de recursos y su relación con el acceso a la atención médica. Como resaltó el destacado sanitarista y bioeticista italiano Giovanni Berlinguer^16^:

“La reflexión ética nos obliga a elegir. Nos obliga a buscar, entre las diversas soluciones posibles, aquellas que se ajustan no solo a criterios de eficiencia y eficacia, equilibrio entre costos y beneficios, sino también a exigencias de prioridad, equidad y moralidad”.^16^

En América Latina, en general, y en Brasil, en especial, la relación entre la bioética y la salud pública/colectiva ha estado presente desde los primeros momentos de su desarrollo, contribuyendo significativamente a ampliar su alcance conceptual y práctico, yendo más allá de los temas biomédicos-biotecnológicos e incorporando cuestiones sanitarias, sociales y ambientales. Bajo la influencia de Berlinguer, quien mantuvo una activa colaboración académica con el país y que en los últimos 30 años de su vida se dedicó a la bioética, un grupo de investigadores brasileños en salud pública optó por seguir este nuevo camino. Las reflexiones presentadas en este estudio, que exploran la relación entre la bioética y el derecho al acceso a la salud, han recibido una atención especial de la bioética brasileña desde entonces^21^^,^^22^^,^^23^^,^^24^^,^^25^^,^^26^^,^^27^^,^^28^.

## CONSIDERACIONES FINALES

Mientras diferentes medios de comunicación de todo el mundo difunden diariamente nuevos descubrimientos y avances extraordinarios en los campos científicos y tecnológicos de la salud, el mundo sigue lidiando con los mismos problemas de salud que han existido durante décadas e incluso siglos, principalmente en las naciones del hemisferio sur, aunque situaciones similares también se registran en las periferias de las grandes ciudades del hemisferio norte. Estos problemas, si bien han disminuido cuantitativamente en los últimos años, aún están lejos de encontrar una solución que sea compatible con los niveles mínimos de respeto a la dignidad humana. Mientras los habitantes de algunas partes del planeta están acostumbrados al acceso a tecnologías de alta complejidad, como los trasplantes múltiples de órganos o el uso clínico de células madre embrionarias, miles de personas adultas, niños y niñas siguen muriendo diariamente en otros lugares debido a problemas simples que podrían haberse evitado mediante medidas sanitarias básicas.

Frente a contextos como este, la humanidad oscila entre la esperanza y la repugnancia; la esperanza de que la economía, la ciencia y la tecnología médica puedan superar estas inaceptables situaciones para que el mundo pueda dejar atrás la contradicción de estar con un pie en el siglo XIX y otro en el siglo XXI; y la repugnancia ante la persistente convivencia cotidiana con tantos niños y niñas, mujeres y personas ancianas en todo el mundo que carecen de acceso a cualquier tipo de atención médica^29^^,^^30^.

Por lo tanto, los conflictos bio(éticos) generados por la contradicción entre la evolución del mundo contemporáneo, el progreso científico y tecnológico en el campo biomédico y el compartimiento y acceso de las personas a los beneficios de este progreso se vuelven cada vez más delicados. En muchos episodios se ha roto la frágil línea que separa estas situaciones, a menudo de manera poco civilizada, en diferentes partes del mundo. Esto crea distorsiones, como la inaccesibilidad para los sectores más empobrecidos de la población, no solo en referencia a los beneficios del desarrollo técnico-científico, sino también en cuanto al acceso a los elementos básicos de consumo sanitario necesarios para una vida digna, incluido el acceso a la atención médica.

No se puede dejar de reforzar una vez más que, en gran medida, gracias al conocimiento científico, el siglo XX prácticamente duplicó la expectativa de vida promedio de las personas en el planeta, lo que generó contingentes numéricamente más representativos de personas de edad avanzada y, por lo tanto, necesitadas de atención geriátrica especializada. Además, los avances relacionados con los nuevos descubrimientos permiten que las personas con acceso a estas nuevas tecnologías puedan vivir más tiempo. Todo esto permite que grupos significativos de la población añadan más años a sus vidas, agregando también más calidad de vida a los años que les quedan. Sin embargo, este nuevo y positivo panorama genera un impacto económico sin precedentes en el acceso a la atención médico-sanitaria, con un aumento significativo de los gastos en el sector.

Para abordar de manera más adecuada toda esta gama de cuestiones, es más acertado que cada país o región del mundo interprete y actúe en sus problemas o situaciones moralmente conflictivas con más justicia y equilibrio, de acuerdo con sus propios contextos sociales, culturales, económicos y biológicos. Algunos movimientos políticos y sociales, como los relacionados con el feminismo, las identidades raciales y étnicas y las diversidades sexuales y de género, entre otros, están logrando mostrar de manera directa y clara a la sociedad y al mundo la importancia de comprender y respetar no solo las diferencias, sino también la fuerza de las acciones colectivas justas. El concepto de diferencia, en cuestiones de género al igual que en cuestiones raciales o de diversidad sexual, no puede significar desigualdad (o inferioridad). Al contrario, debe recuperar la necesidad democrática de que cada situación se contextualice exactamente a partir de estos parámetros diferenciales para buscar la verdadera igualdad. De la misma manera, estos grupos organizados con objetivos claros a través de acciones orgánicas están ganando cada vez más atención mediática y poder de presión pública sobre los gobiernos para lograr avances en el espacio social.

En resumen, desde aspectos más simples y directos como los derechos de una mujer embarazada, hasta aquellos que se refieren a la igualdad de acceso a la atención médica para todas las personas sin distinción, conquistas consideradas distantes para la gran mayoría de la población en países empobrecidos, pero también para algunos grupos excluidos en países enriquecidos, a pesar de las dificultades que aún enfrentan, pueden recibir nuevos impulsos a partir de presiones sociales y políticas desarrolladas por estos movimientos democráticos, sumados a posibles avances morales en el comportamiento gubernamental (aunque se conocen las dificultades en este sentido) y de la propia sociedad global.

El propósito de este texto, por lo tanto, ha sido mostrar que el acceso a la atención médica para todas las personas, independientemente de su nivel de ingresos, debe considerarse como un derecho humano universal no negociable, que además de las obligaciones de los gobiernos y del sector privado de respaldar programas inclusivos, también debe considerar legítima la lucha de diversos movimientos sociales en defensa de mejores condiciones de vida y salud para todas las personas sin distinción.
